# Morphological and molecular identification of nasopharyngeal bot fly larvae infesting red deer (*Cervus elaphus*) in Austria

**DOI:** 10.1007/s00436-016-5206-z

**Published:** 2016-08-05

**Authors:** Natascha Leitner, Laurin Schwarzmann, Carina Zittra, Nicola Palmieri, Barbara Eigner, Domenico Otranto, Walter Glawischnig, Hans-Peter Fuehrer

**Affiliations:** 1 Institute of Parasitology, Department of Pathobiology, University of Veterinary Medicine Vienna, Veterinaerplatz 1, 1210 Vienna, Austria; 2 Department of Veterinary Medicine, University of Bari, Str. Prov. Casamassima km3, 70010 Valenzano, Bari Italy; 3 Institute for Veterinary Disease Control Innsbruck, Austrian Agency for Health and Food Safety Ltd. (AGES), Technikerstrasse 70, 6020 Innsbruck, Austria

**Keywords:** *Cephenemyia auribarbis*, *Cervus elaphus*, Cytochrome oxidase subunit I, Myiasis, Oestridae, *Pharyngomyia picta*

## Abstract

Nasopharyngeal myiases are caused by larvae of bot flies (Diptera: Oestridae), which have evolved a high specificity for their hosts. Bot flies (*n* = 916) were collected from 137 (57.6 %) out of 238 red deer (*Cervus elaphus*) hunted in Vorarlberg and Tyrol (Western Austria). After being stored in 75 % ethanol, larvae were identified to species level and developmental stage using morphological and morphometric keys. Larvae were also molecularly characterized by polymerase chain reaction (PCR) amplification and partial sequencing of the mitochondrial cytochrome oxidase subunit I gene. Morphological and molecular analysis allowed identification of larvae as C*ephenemyia auribarbis* and *Pharyngomyia picta*. Genetic variations were also examined within the specimens collected in both geographical locations.

## Introduction

To date, nasopharyngeal bot flies have been mostly studied in relationship to the economic damage they cause in animal husbandry (Otranto et al. 2003). Nasopharyngeal bot flies are known to be host specific and to cause obligatory myiasis in a wide range of animal species (Catts and Mullen 2002). Their development is holometabolic but (compared to other insects) is characterized by being larviparous (Catts and Mullen 2002; Colwell 2001). Soon after adult flies spray the first-stage larvae into the nostrils of the host, larvae migrate into the nasal and sinus cavities of the pharynx (Colwell 2001) therefore causing irritation, rhinitis, nasal discharge, purulent mucous exudates, and respiratory problems (Catts and Mullen 2002). Red deer (*Cervus elaphus*) is the main host for the nasopharyngeal bot fly species *Cephenemyia auribarbis* and *Pharyngomyia picta* (Colwell 2001; Vicente et al. 2004), both belonging to the subfamily Oestrinae (Catts and Mullen 2002; Colwell 2001). In addition, *Cephenemyia stimulator*, which is adapted to roe deer (*Capreolus capreolus*) (Colwell 2001), has on occasion been diagnosed in red deer (Király and Egri 2004, 2007). While the host spectrum of *P. picta* is broader, as this species can be detected regularly in red deer, sika deer (*Cervus nippon*), fallow deer (*Dama dama*), and roe deer, *C. auribarbis* is usually found in red deer and fallow deer only (Colwell 2001). Previous studies have shown that *C. auribarbis* occurs less frequently in red deer compared to *P. picta* (summarized in Vicente et al. 2004). Mixed infections of both *C. auribarbis* and *P. picta* in the same individual host were reported in Hungary (Sugár 1974) and in Spain (Ruíz Martinez and Palomares 1993), in 71.5 and 23.1 % of examined red deer, respectively.

Molecular tools have shown to be successful and reliable for diagnostic and taxonomic studies of oestrid larvae (Otranto and Stevens 2002), and in particular, the cytochrome oxidase subunit I (*cox*I) mitochondrial gene has been used in several studies for the diagnosis and phylogeny of larvae within this taxon (Otranto and Stevens 2006).

Although the majority of bot fly infections occur in wildlife hosts, there is a lack of knowledge regarding these oestrids (Colwell et al. 2009). The aim of this study was not only to examine the species diversity of bot flies in Western Austrian red deer but also the use of morphometric and molecular tools for the analysis of variations (inter- and intra-species) within populations of these oestrids in a limited regional area.

## Materials and methods

During the course of a regular shooting program, 238 red deer were shot in Western Austria (183 in Vorarlberg and 55 in Tyrol) from April to July 2014 and sent to the AGES Institute for Veterinary Disease Control in Innsbruck, Austria, immediately for necropsy. Sampling location, date, and sex of the red deer were collected whenever possible. Nasopharyngeal bot fly larvae were sampled at necropsy, stored in 75 % ethanol at room temperature, and sent to the University of Veterinary Medicine Vienna, Austria, for further analysis. Bot fly larvae were cleaned from host nasal secretion using soft toothbrushes and microscopically identified to species level and developmental stage using identification keys by Zumpt (1965), Sugár (1974), and Draber-Monko (1975). Thirteen larval samples from ten red deer were sent to the Department of Veterinary Medicine, University of Bari, Italy, for blinded species identification.

A subset of the specified bot fly larvae were further analyzed by additional morphological tools and molecular techniques. Larvae (*n* = 48; *C. auribarbis* L3 = 11 and L2 = 3 and *P. picta* L3 = 30 and L2 = 4) were measured with regard to the following: body length, from the tip to the end of the larva; body width at the widest part of the larva; shape of posterior spiracular plates; and distance between the antennal lobes at the base of the antenna (Nikon SMZ 1270 and NIS Elements D 4.30.02-software, Nikon, Tokyo, Japan).

DNA was extracted from 44 larvae (10 *C. auribarbis* and 34 *P. picta*) using the DNeasy™ Blood and Tissue Kit (Qiagen, Hilden, Germany) according to the manufacturer’s instructions. PCR amplifications were performed with dipteran-specific barcode primers (H15CuliCOIFw:5′-AGCCATTTAATCGCGACAA-3′;H15CuliCOIRv:5′-GGATGTCCAAAAAATCAAAATAAATGTT-3′) in a Mastercycler Gradient S (Eppendorf, Germany) under conditions reported previously (Zittra et al. 2016). Amplification products were subjected to agarose gel electrophoresis on 1.8 % agarose gels at 120 V for 70 min. Subsequently, gels were stained with Midori Green Advance (Biozym Scientific GmbH, Hess. Oldendorf, Germany), and PCR amplicons of approximately 730 bp were visualized by UV light. DNA sequencing was performed at LGC GmbH (Berlin, Germany). All phylogenetic analyses were conducted with MEGA6 (Tamura et al. 2013), and sequences were aligned with ClustalW (Thompson et al. 1994) including *Drosophila melanogaster* as the out-group. The GTR model of sequence evolution with uniform rates was chosen by scanning 24 possible models and selecting the one with the lowest Bayesian information criterion. The tree was constructed by maximum likelihood using 1000 bootstrap replicates.

## Results and discussion

**Fig. 1:**
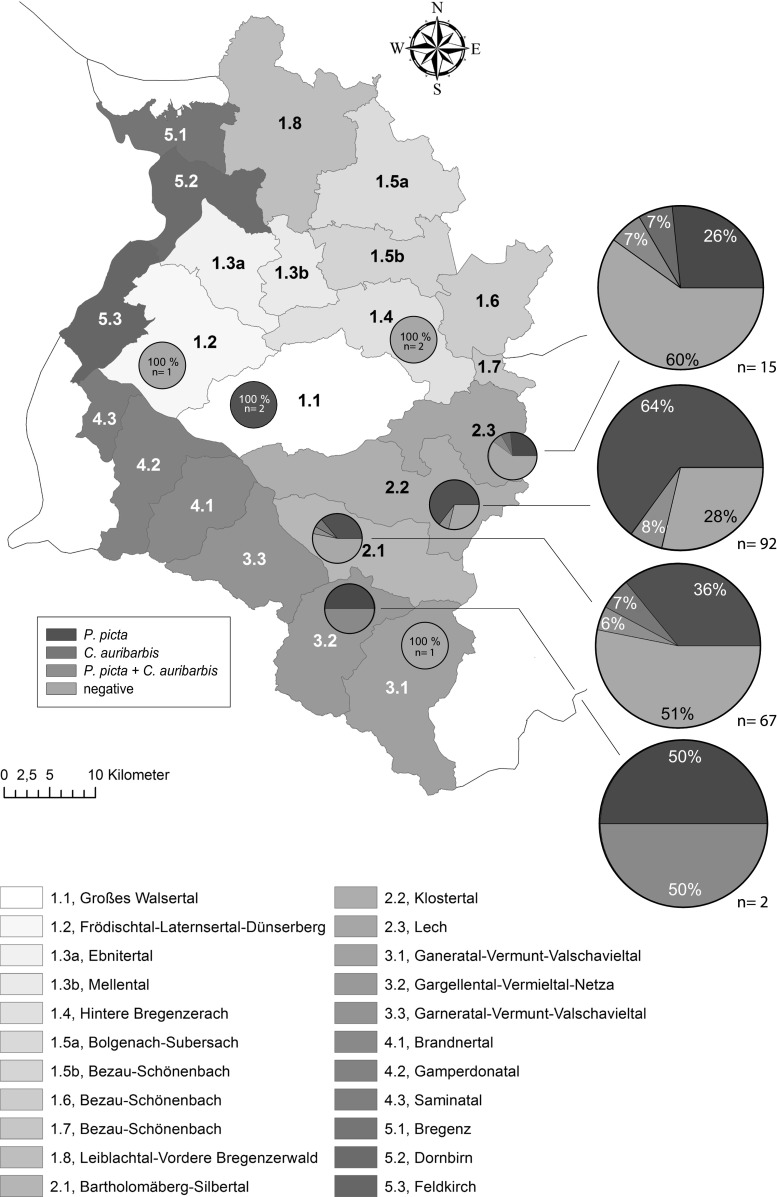
Distribution of larval species per host in Vorarlberg (*n* = 110 red deer hosts in Vorarlberg; ESRI ArcGIS 10.1® was used for graphic design)

**Fig. 2:**
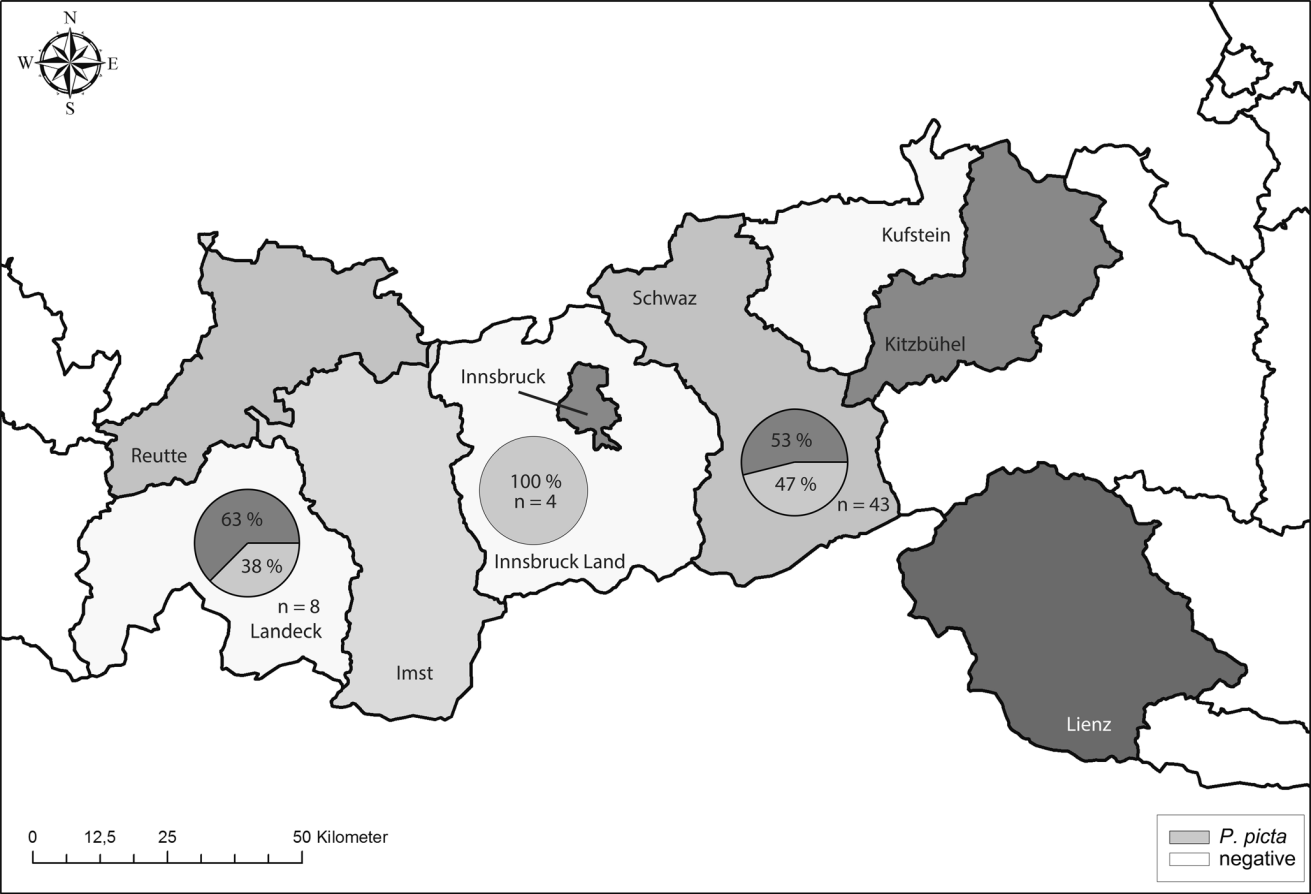
Distribution of pharyngeal bot flies in red deer in Tyrol (*n* = 55 red deer hosts in Tyrol) per district (ESRI ArcGIS 10.1® was used for graphic design)

Overall a total of 916 bot fly larvae—49 were identified as *C. auribarbis* and 827 as *P. picta*—were retrieved in 137 out of 238 red deer (57.6 %; 95 %CI, 51.2–63.7 %; Figs. 1 and 2). For 40 larval specimens, the morphological identification was not possible due to their poor condition. *P. picta* was documented in 130 red deer (54.6 %; 95 %CI, 48.3–60.8 %) whereas *C. auribarbis* was found in 19 red deer (8 %; 95 %CI, 5.2–12.1 %). Co-infections with both bot fly species were detected in 13 individual hosts (5.5 %; 95 %CI, 3.2–9.1 %). Both prevalence and distribution varied within the provinces sampled in Vorarlberg and Tyrol. *C. auribarbis* was only found in animals from Vorarlberg whereas *P. picta* was the prominent bot fly species in red deer in both regions (Vorarlberg, 56.3 %; Tyrol, 49.1 %). The prevalence of nasal bot fly in red deer is known to vary from 41.89 % in central Spain (De la Fuente et al. 2000) to 85 % in Spain (Bueno-de la Fuente et al. 1998), 90 % in southern Spain (Ruíz Martinez and Palomares 1993), and 98.2 % in Hungary (Sugár 1974). Our findings are in general agreement with these previous studies though the sampling season may affect the prevalence of bot flies in red deer and also the co-occurrence of both *C. auribarbis* and *P. picta* in the same host (cf. Vicente et al. 2004; Ruíz Martinez and Palomares 1993). Overall, *P. picta* was the dominant species found in this study, whereas *C. auribarbis* was rarely identified in infested animals in Vorarlberg and not in Tyrol. This may be related to the different developmental periods throughout the year (cf. Vicente et al. 2004; Ruíz Martinez and Palomares 1993). Concerning the seasonal dynamics in Vorarlberg, the number of larvae per host as well as the prevalence decreases throughout the sampled period from April to June (Table 1). Similar to the findings of Ruíz Martinez and Palomares (1993), no differences in the host preference between male and female infected hosts were observed in this study. The data conflict with previous observations by Vicente et al. (2004) and Bueno-de la Fuente et al. (1998) who recorded a higher prevalence in male hosts.

**Table 1:** Prevalence and mean number of larvae per host per sampled month (2014) and region

	Positive hosts	Total hosts	Prevalence (%)	Mean larvae
Vorarlberg				
April	24	27	88.9	9.8
May	54	88	61.4	5.9
June	32	68	47.1	4.1
Tyrol				
May	15	20	75.0	6.7
June	8	20	40.0	12.3
July	4	15	26.7	4.5

The retrieval of the majority of L3 and any L1 larvae might be associated with the sampling season (the percentages of larval stages which could be clearly identified were *P. picta*: *n*_L2_ = 9.1 %, *n*_L3_ = 90.9 %; *C. auribarbis*: *n*_L2_ = 8.7 %, *n*_L3_ = 91.3 %). Morphometric analysis of the bases of the antennal lobes allowed differentiation of *C. auribarbis* L3 (mean value = 0.187 mm, *σ* = 0.08) from *P. picta* L3 (mean value = 0.482 mm, *σ* = 0.07). The mean body length of *C. auribarbis* L3 was 29.391 mm (*σ* = 2.21), compared to 24.939 mm (*σ* = 2.59) in *P. picta* (Table 2). For the morphological analysis, the general shape (especially the posterior spiracles) of the larvae, but also body length, dorsal spine rows, and pigmentation allowed species differentiation (cf. Zumpt 1965; Sugár 1974; Draber-Monko 1975).

**Table 2:** Mean body width (mm) and body length (mm) per bot fly species and larval stage

Body width and length (mm)	L2	L3
*P. picta* body width	5.368 (*σ* = 2.50)	7.895 (*σ* = 0.56)
*P. picta* body length	17.534 (*σ* = 5.49)	24.939 (*σ* = 2.59)
*C. auribarbis* body width	5.364 (*σ* = 0.56)	7.489 (*σ* = 0.54)
*C. auribarbis* body length	16.009 (*σ* = 1.75)	29.391 (*σ* = 2.21)

**Fig. 3:**
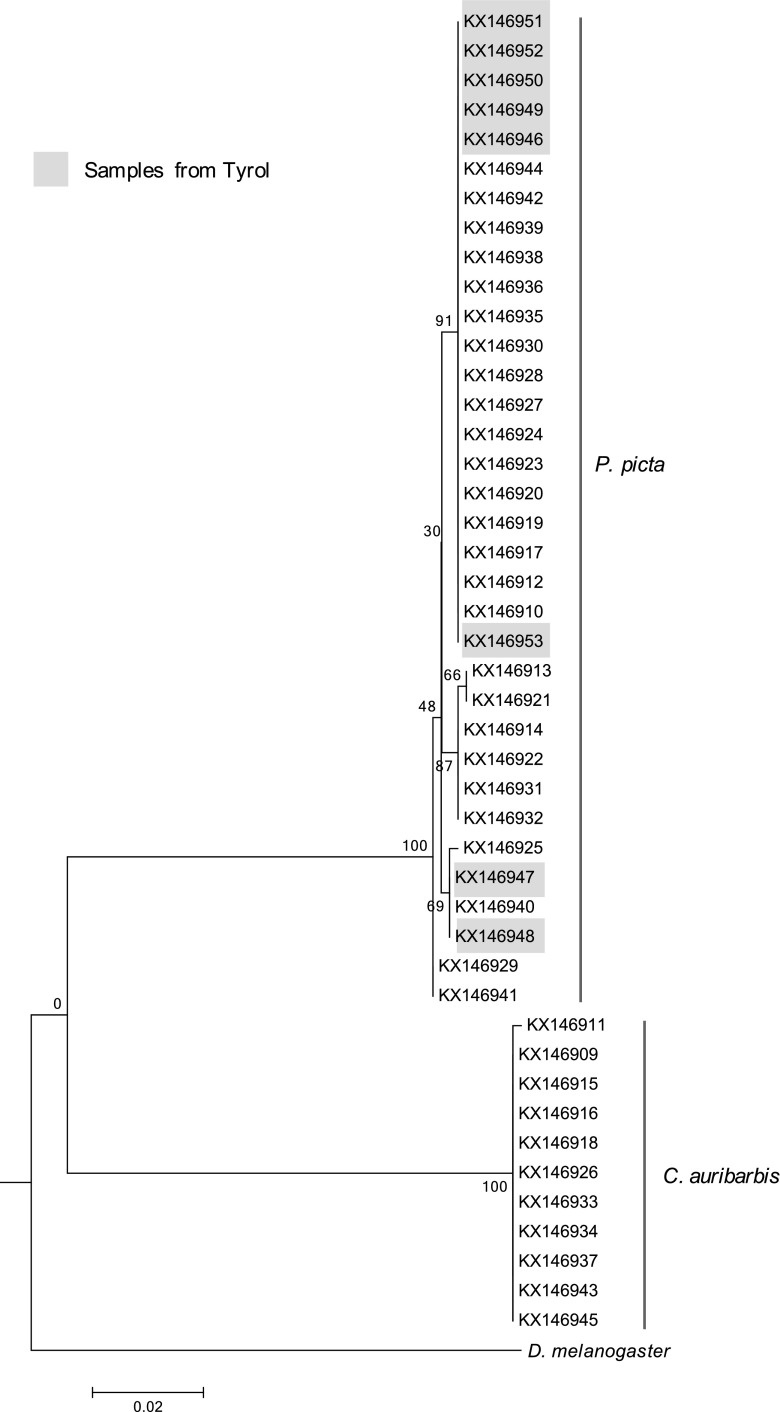
Molecular phylogenetic analysis by maximum likelihood method. The evolutionary history was inferred by using the maximum likelihood method based on the general time reversible model (Nei and Kumar 2000). The tree with the highest log likelihood (−1580.9403) is shown. The percentage of trees in which the associated taxa clustered together is shown next to the branches. Initial tree(s) for the heuristic search were obtained automatically by applying neighbor-joining and BioNJ algorithms to a matrix of pairwise distances estimated using the maximum composite likelihood (MCL) approach and then selecting the topology with superior log likelihood value. The tree is drawn to scale, with branch lengths measured in the number of substitutions per site. The analysis involved 46 nucleotide sequences. There were a total of 679 positions in the final dataset. Evolutionary analyses were conducted in MEGA6 (Tamura et al. 2013)

Nucleotide sequences of 44 bot fly larvae (10 *C. auribarbis* and 34 *P. picta*) were analyzed within the barcode region of the cytochrome oxidase subunit I gene. *C. auribarbis* and *P. picta* could be separated clearly showing variations in 87 loci (GenBank™: KX146909-KX146953). Furthermore intra- and inter-species variations were observed within the barcode region of *C. auribarbis* and *P. picta* (Fig. 3) therefore indicating that the selected target gene is effective for the species discrimination. However, further molecular-based studies are needed to monitor genetic variation within the populations of bot flies in Austria.
